# On fractality of functional near-infrared spectroscopy signals: analysis and applications

**DOI:** 10.1117/1.NPh.7.2.025001

**Published:** 2020-04-29

**Authors:** Li Zhu, Sasan Haghani, Laleh Najafizadeh

**Affiliations:** a Rutgers University, Integrated Systems and NeuroImaging Laboratory, Department of Electrical and Computer Engineering, Piscataway, New Jersey, United States; b University of The District of Columbia, Department of Electrical and Computer Engineering, Washington DC, United States

**Keywords:** functional near-infrared spectroscopy, optical brain imaging, visibility graph, fractal dimension, resting state, classification, brain computer interfaces

## Abstract

**Significance:** The human brain is a highly complex system with nonlinear, dynamic behavior. A majority of brain imaging studies employing functional near-infrared spectroscopy (fNIRS), however, have considered only the spatial domain and have ignored the temporal properties of fNIRS recordings. Methods capable of revealing nonlinearities in fNIRS recordings can provide new insights about how the brain functions.

**Aim:** The temporal characteristics of fNIRS signals are explored by comprehensively investigating their fractal properties.

**Approach:** Fractality of fNIRS signals is analyzed using scaled windowed variance (SWV), as well as using visibility graph (VG), a method which converts a given time series into a graph. Additionally, the fractality of fNIRS signals obtained under resting-state and task-based conditions is compared, and the application of fractality in differentiating brain states is demonstrated for the first time via various classification approaches.

**Results:** Results from SWV analysis show the existence of high fractality in fNIRS recordings. It is shown that differences in the temporal characteristics of fNIRS signals related to task-based and resting-state conditions can be revealed via the VGs constructed for each case.

**Conclusions:** fNIRS recordings, regardless of the experimental conditions, exhibit high fractality. Furthermore, VG-based metrics can be employed to differentiate rest and task-execution brain states.

## 1 Introduction

Functional near-infrared spectroscopy (fNIRS) is a promising noninvasive imaging technique that uses light in the near-infrared range to measure the local changes in the oxy-($\Delta [{\mathrm{HbO}}_{2}]$) and deoxygenated hemoglobin (Δ[HbR]) concentrations associated with the underlying brain activities.^1^ Compared to functional magnetic resonance imaging (fMRI), which is only sensitive to Δ[HbR], fNIRS provides additional information related to brain activity by also measuring $\Delta [{\mathrm{HbO}}_{2}]$. Additionally, fNIRS is portable and relatively less expensive compared to most neuroimaging modalities, enabling studying the brain function in more realistic settings.^2^^–^^6^ Because of these advantages, fNIRS has been used in several cognitive and clinical neuroscience studies^7^^–^^14^ as well as those involving brain–computer interfaces (BCIs).^15^^–^^19^

A majority of fNIRS brain imaging studies have focused on the spatial domain and typically have ignored consideration of changes that occur in the temporal domain. Examples include localization studies,^20^^–^^22^ where the aim is to identify brain’s activation patterns in response to specific stimuli, and connectivity studies (functional or effective), where the focus is on investigating the functional interactions among brain regions, either when the brain is at rest or is engaged in performing a particular task.^23^^–^^27^ However, it is now well known that the brain is highly dynamic,^28^^–^^32^ and therefore, to gain a more comprehensive picture about its function, methods capable of extracting temporal information in brain recordings are required.

As compared to the spatial domain, a much smaller number of fNIRS studies exists that have considered the temporal domain for the analysis.^33^^–^^40^ For example, in Ref. 33, by applying the Higuchi fractal dimension algorithm,^41^ it is shown that fNIRS signals have high degree of complexity. The wavelet transform is applied to fNIRS signals, and it is shown that the wavelet coefficients can be used to train a classifier. In Refs. 38–40, entropy has been used to assess the complexity of fNIRS signals in patient groups (such as those with Alzheimer’s disease, attention-deficit hyperactivity disorder, and traumatic brain injury), demonstrating that it carries information that can be related to diseases. All these studies suggest that there exists information relevant to the underlying brain activity in the complex characteristics of fNIRS signals.

In this paper, using visibility graph (VG), we present an approach for revealing the fractal properties of fNIRS time series. VG is a recently introduced method, which maps a time series into a graph (called a VG). As will be discussed, the topological properties of the constructed graph are related to the fractality and complexity of the time series.^42^^,^^43^ Compared to conventional fractal analysis approaches,^42^ VG is computationally less complex and has been used in various studies.^44^^–^^49^ For example, using electrocardiogram, Jiang et al. showed that employing VG analysis can reveal the dynamical changes caused by mediation training, manifested as regular heartbeat, which is closely related to the adjustment of the autonomous neural system.^44^ Zhu et al. applied a VG-based approach for alcoholism identification, showing that this approach is promising in separating alcoholic subjects from controlled drinkers.^48^ In Ref. 47, it was shown that VG applied to electroencephalography (EEG) signals can provide features that can distinguish children with autism from nonautistic children. In Ref. 49, we have shown that the temporal characteristics of calcium recordings in GCaMP6 mice extracted by VG carries discriminatory information that can be utilized to decode behavior.

**Fig. 1:**
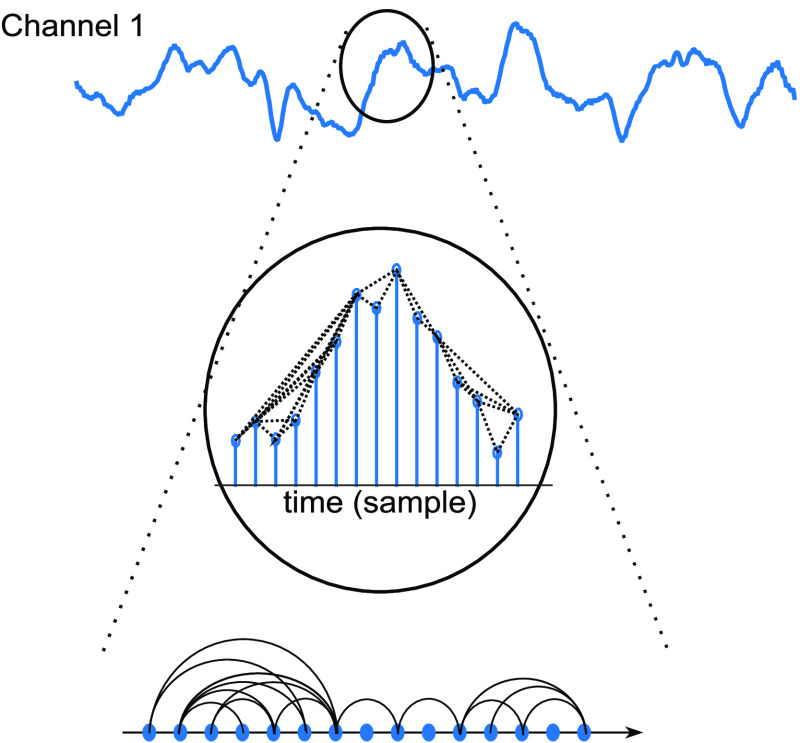
Conceptual illustration of constructing VG from a time series. Nodes in the graph correspond to the time points in the time series, and links are formed based on the visibility condition.

It is important to note here the difference between VG and the graph theoretical-based methods used commonly in functional connectivity studies.^50^^,^^51^ In typical functional connectivity studies, graphs are constructed in the spatial domain, i.e., nodes in the graph correspond to the location of channels or voxels, and links between two nodes are formed based on the statistical similarity of the time series associated with the two nodes, quantified by measures, such as correlation. On the other hand, as will be discussed in Sec. 2, in VG, the nodes correspond to the time points in the time series, and the links are formed based on natural visibility between the time points (Fig. 1). Once the graph is formed for each time series, graph measures can be extracted to represent different properties of the time series.

In this paper, we use VG to study the fractality of fNIRS time series under two conditions: when the brain is at rest and when it is engaged in the task. fNIRS time series are recorded from nine healthy male subjects at two resting-state conditions and two task conditions. VGs are constructed from each time series for each channel and each condition. The power of scale-freeness of visibility graph (PSVG) is then extracted and compared across conditions.

To the best of our knowledge, this is the first study to employ VG to reveal temporal characteristics of fNIRS-recorded time series, demonstrating its feasibility in identifying features in fNIRS recordings that can be used to gain new insights about the brain function.

The rest of this paper is organized as follows. Section 2 describes the methods used for analysis in this study. The details of the experimental setup are given in Sec. 3. Section 4 presents the results, and finally, some discussions are provided in Sec. 5.

## 2 Methods

Fractal analysis of time series provides an interesting opportunity to study their temporal structure in terms of self-similarity, power-law scaling relationship, and scale-invariance.^52^ Since its introduction, fractal analysis has been used in several fields, such as physics, geography, biology, and psychology^53^ to reveal properties in time series that will not be visible through conventional analysis. Recently, fractal analysis has also been applied to recordings obtained through functional brain imaging studies^42^^,^^54^^–^^56^ to better characterize the observed temporal fluctuations of the signals or to differentiate diseased group from healthy population with an improved accuracy. For instance, using fractal analysis of fMRI data, Sokunbi et al. showed that the hemodynamic response measured from patients with Schizophrenia presented larger complexity, compared to that measured from healthy controls.^57^ He showed that the fractal properties of the fMRI signal altered with the changes of brain functional state.^58^ Using fNIRS, Khoa and Nakagawa showed that the complex characteristics of the signals recorded during physical motion and imaginary motion of the right arm were different, and these differences can be potentially used in BCIs.^33^ Lei et al. showed that the power spectra of both EEG and fMRI signals follow the power-law distribution, and scale-free brain activity can be characterized by robust temporal structures.^59^

There exist several methods to estimate the fractality of a time series,^52^ and the choice of the proper method has to be made based on the properties of the time series, and whether it is stationary or nonstationary. In this paper, the fractal properties of fNIRS time series will be evaluated using two approaches: scaled windowed variance (SWV) analysis and VG. These methods are described here.

In the following, we denote an N-point time series (e.g., recorded by a given fNIRS channel) with $x={\left\{x_{i}\right\}}_{i=1}^{N}$.

### 2.1 Scaled Windowed Variance Analysis

We first evaluate the fractal dimension of fNIRS time series using conventional methods. As it is well known that physiological signals and brain activities are nonstationary,^52^^,^^60^ SWV analysis method^52^^,^^61^ is used here to estimate the fractal dimension (defined below) of fNIRS-recorded time series.

To estimate the fractal dimension of a time series using SWV, the time series is partitioned into nonoverlapping segments of size n. If $\hat{\mu }$ represents the mean of the segment, the standard deviation for each segment, σ^n, is computed as follows:^52^

σ^n=1n−1∑i=1n(xi−μ^)2. (1)

This measure is computed for all segments and then is averaged to obtain ${\overset{¯}{\sigma }}_{n}$. The procedure is repeated for different window sizes.

For nonstationary fractal time series, the windowed mean standard deviation, ${\overset{¯}{\sigma }}_{n}$, and its corresponding window size n follow a power law relation given by^52^

$${\overset{¯}{\sigma }}_{n}\overset{d}{=}p\cdot n^{H},$$ (2)

where $\overset{d}{=}$ represents equal in distribution, H is the Hurst coefficient that can be obtained by calculating the slope of the least-squares linear regression line of $\log({\overset{¯}{\sigma }}_{n})$ versus log(n), and p is a proper prefactor.^52^ The fractal dimension, D, is linearly related to H as D=2−H.^62^

### 2.2 Visibility Graph

VG is a recently introduced method for studying the fractal properties of time series.^43^ The VG associated with a given time series x of N points is constructed as follows (see Fig. 1). Each time point in x is considered as a node in the graph (i.e., for an N-point time series, the graph will have N nodes). The link between two nodes is formed if the nodes are considered to be “naturally visible.” That is, in the graph, there will be a link between nodes h and l (corresponding to time points $t_{h}$ and $t_{l}$ in the time series), if and only if, for any node p ($t_{h}<t_{p}<t_{l}$), the following condition holds:^43^

$$x_{p}<x_{l}+[x_{h}-x_{l}][\frac{t_{l}-t_{p}}{t_{l}-t_{h}}].$$ (3)

The VG is expressed using the adjacency matrix, an N×N symmetric matrix $A_{N\times N}=[a_{hl}]$, where $a_{hl}=1$ when nodes h and l are connected and $a_{hl}=0$, otherwise.^42^

The graph that is constructed through VG reveals the dynamic properties of the time series in unique ways. For example, periodic signals result in regular graphs, and fractal time series result in scale-free networks.^42^^,^^43^^,^^45^^,^^63^ Scale-free corresponds to the property of the graph that, independent of the number of nodes, its degree distribution P(k) has a power-law tail, where the tail exponent obeys the power law, i.e.,

$$P(k)∼k^{-\gamma }.$$ (4)

In Eq. (4), k represents the degree of the node (i.e., the number of links connected to a node), P(k) denotes the degree distribution (i.e., the fraction of nodes with degree k), and γ denotes the PSVG. It has been proven that the PSVG is indicative of the fractality of the time series.^64^

## 3 Experimental Procedure

**Fig. 2:**
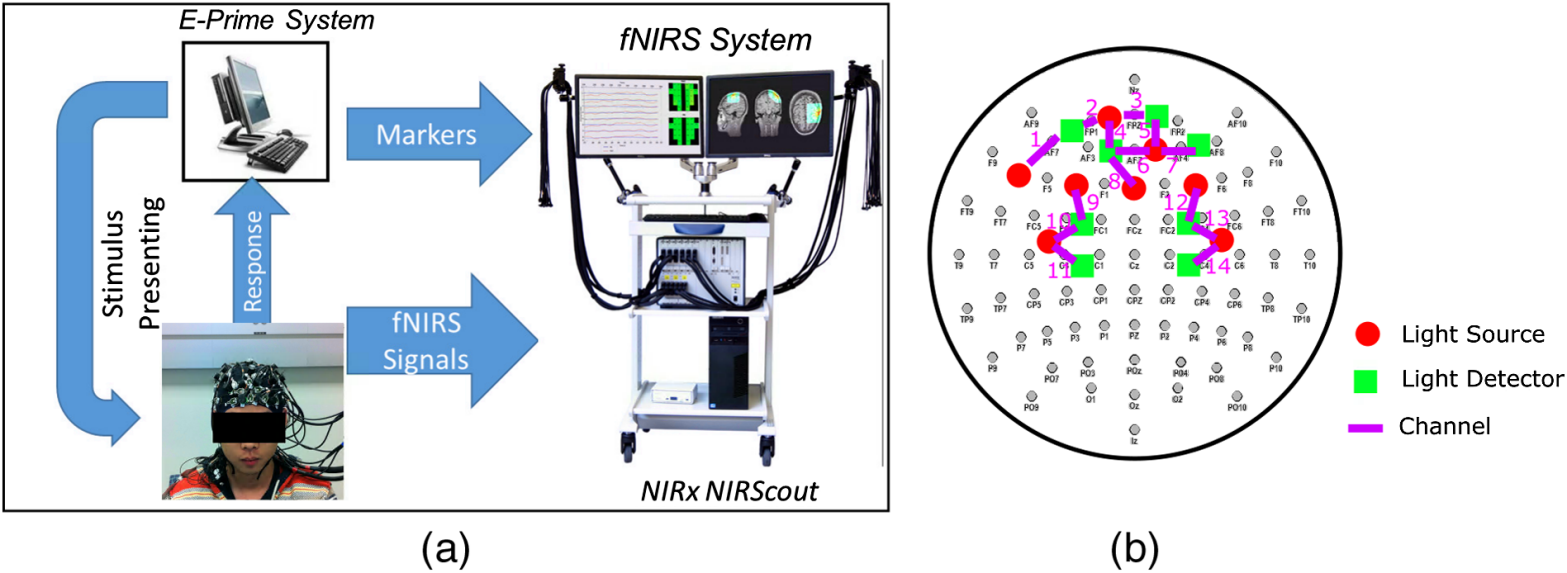
(a) Illustration of the experimental setup and (b) map of fNIRS optode locations. Optodes are placed to cover the prefrontal and motor cortices. For each channel, the distance between the light source and the light detector is 3 cm.

In this section, we describe the experimental setup and the preprocessing steps used to remove artifacts from fNIRS signals. Figure 2 shows the experimental setup and the location of optodes.

### 3.1 Experimental Paradigm and Data Acquisition Procedure

Nine healthy male subjects (age mean: 25, age SD: 4.8) participated in our experiments, after providing their written informed consents, approved by the Rutgers Institutional Review Board. The experiments included two resting-state sessions [eyes-closed (EC) and eyes-opened (EO)] and two block-designed (number of blocks = 3) tasks. Each resting-state session lasted for 10 min. The tasks were the response time (RT) task and the modified Go No-Go (GNG) task. Each block in RT task consisted of 50 left and 50 right arrows presented to participants in random order with intertrial interval (ITI) of [800 to 1200] ms. Participants were asked to press right/left mouse button depending on the direction of presented arrow. Each block in the modified GNG task consisted of 60 Go and 30 No-Go symbols with ITI of [800 to 1200] ms.^65^ Participants were asked to click only when the Go symbol is presented. Recordings for the two resting-state and the two tasks sessions were all completed in one setting.

Changes in the optical signals were recorded at two wavelengths (685 and 830 nm) at the sampling rate of 12.5 Hz, using NIRScout system (NIRx Medical Technologies, LLC). Note that although the maximum sampling rate of NIRScout system is 62.5 Hz, in this experiment, 12.5 Hz was used as the practical sampling rate due to the time-division multiplexing on the illuminated light sources to avoid cross talk. A total of 14 channels were used (8 light sources and 8 detectors arrangement). For each channel, the distance between the light source and the light detector was 3 cm. No short distance probes^66^ were used. Optodes were placed to cover the prefrontal and motor cortices, where brain activities are expected for these tasks according to prior studies.^65^^,^^67^^,^^68^

### 3.2 Data Preprocessing

In this section, we describe the artifact rejection, bandpass filtering, and hemodynamic signal conversion procedures.

Motion-related artifacts inevitably exist in fNIRS-recorded time series, and if not removed, can negatively impact the outcome of fractal analysis. Therefore, artifact rejection procedure was first performed.^69^ Two types of artifacts, “spikes” and “discontinuities” (jumps), were particularly considered, as they could impact the construction of VG. For each channel, short intervals containing spikes were identified by visual inspection and then replaced by the average of the signals of the same interval from two neighboring channels. Discontinuities were detected by examining the difference in signal values at every two successive time points. For every time point pair, discontinuity was detected if this difference becomes larger than four times of the standard deviation of the time series. In such cases, the discontinuity was eliminated by subtracting the difference from the values for the point after the jump.^70^

Next, optical signals were band-pass filtered using a fourth-order band-pass Butterworth filter in the range of 0.01 to 0.2 Hz to exclude the low-frequency drift and the interference caused by physiological sources, such as heart beat and respiration.^71^^,^^72^ The filtered signals were converted into $\Delta [{\mathrm{HbO}}_{2}]$ and Δ[HbR] according to the modified Beer–Lambert’s law, based on the following equations:^73^

$$\ln(\frac{I_{\lambda_{1}}}{I_{\text{baseline},\lambda_{1}}})=-(\epsilon_{{\mathrm{HbO}}_{2},\lambda_{1}}\cdot \Delta [{\mathrm{HbO}}_{2}]+\epsilon_{\mathrm{HbR},\lambda_{1}}\cdot \Delta [\mathrm{HbR}])\cdot {\mathrm{DPF}}_{\lambda_{1}}\cdot x,\;\ln(\frac{I_{\lambda_{2}}}{I_{\text{baseline},\lambda_{2}}})=-(\epsilon_{{\mathrm{HbO}}_{2},\lambda_{2}}\cdot \Delta [{\mathrm{HbO}}_{2}]+\epsilon_{\mathrm{HbR},\lambda_{2}}\cdot \Delta [\mathrm{HbR}])\cdot {\mathrm{DPF}}_{\lambda_{2}}\cdot x.$$ (5)

Note that $\Delta [{\mathrm{HbO}}_{2}]$ and Δ[HbR] are the unknown parameters here and will be found by solving this system of two linear equations. In Eq. (5), $I_{\lambda_{i}}$ and $I_{\text{baseline},\lambda_{i}}$ (i=1,2) are the optical intensities measured at the detector location at wavelength $\lambda_{i}$ during experimental block and during preblock baseline period, respectively, x is the distance between the light source and the light detector, ${\mathrm{DPF}}_{\lambda_{i}}$ is the differential pathlength factor, and $\epsilon_{{\mathrm{HbO}}_{2},\lambda_{i}}$ and $\epsilon_{\mathrm{HbR},\lambda_{i}}$ are the extinction coefficients of ${\mathrm{HbO}}_{2}$ and HbR at wavelength $\lambda_{i}$, respectively. Here, $I_{\text{baseline},\lambda_{i}}$ for $\lambda_{i}$ is obtained as the averaged recorded light intensity, associated with wavelength $\lambda_{i}$, during the 10-min EC resting-state session.

The artifact rejection, band-pass filtering, and data conversion procedures were performed using the nirsLAB toolbox.^70^^,^^74^ For the RT and GNG tasks, time series were segmented according to the border of the blocks (170 s for each block). The same duration was used to segment the signals from the resting-state recordings into three nonoverlapping blocks. This procedure for all subjects resulted in a total of 108 fourteen-channel time series, each with 2125 data points.

## 4 Results

In this section, we present the results of fractal analysis from the SWV and VG approaches.

### 4.1 Results from Scaled Windowed Variance Analysis

**Fig. 3:**
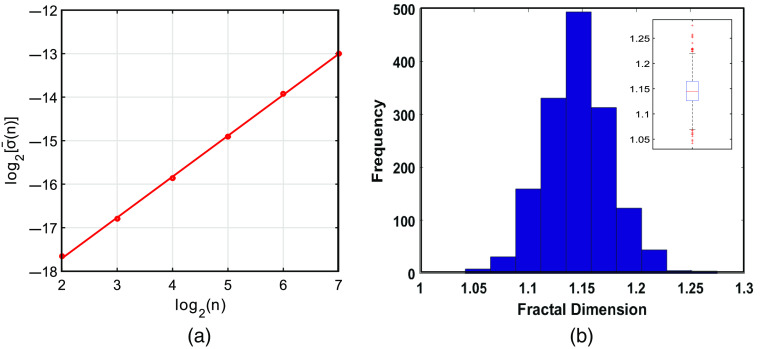
Results from SWV analysis. (a) Calculated windowed mean standard deviation as a function of the window size in a double logarithmic plot for a given time series. The slope of the fitted least square trend is related to the fractal dimension of the time series, and (b) distribution of the estimated fractal dimension for all fNIRS time series.

Fractality of fNIRS recordings, for each channel, subject, condition, and block, was first examined using SWV analysis, which is an appropriate method for nonstationary fractal time series.^52^ Figure 3(a) shows the obtained mean standard deviation [${\overset{¯}{\sigma }}_{x}(n)$] as a function of the window size (n) in a double logarithmic plot for a given time series. As can be seen, for the window size ranging from $2^{2}$ to $2^{7}$, the data follow a linear trend. By definition, the slope of this trend equals the Hurst exponent, H, from which the fractal dimension D can be estimated from D=2−H.^52^ As a measure of self-similarity, the value of D ranges between 1.0 and 2.0. D for a random time series is 1.5. For a time series with high correlation or memory over time, the extracted D value is near 1.^62^

The distribution of estimated D values for all preprocessed time series (resting-state and task) is shown in Fig. 3(b). As can be seen, the estimated D value for all time series is less than 1.3 with a mean of 1.15. This result confirms the existence of high degree of fractality (positive correlation) in fNIRS time series, and hence, VG analysis can be applied to these signals.

### 4.2 Results from Visibility Graph Analysis

**Fig. 4:**
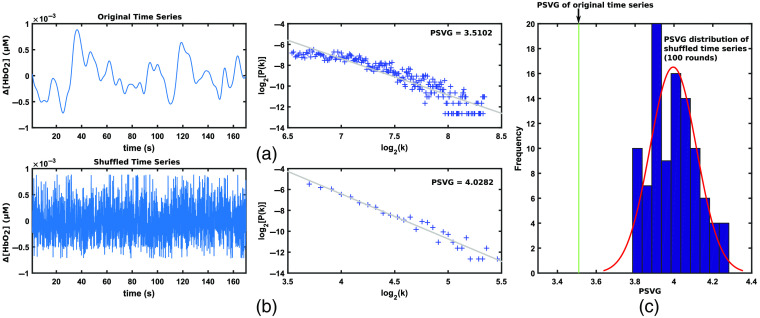
Comparison of the PSVG, estimated for a representative resting-state block of fNIRS time series, and its randomly shuffled version. (a) The time course of a representative fNIRS recording and its power-law tail of the degree distribution, (b) the time course of randomly shuffled version of the same time series and its power-law tail of the degree distribution, and (c) comparison between the PSVG of the original time series and the distribution of PSVGs for 100 randomly shuffled versions of the original time series.

We first examine that the temporal structure of the fNIRS signals can be characterized using the PSVG measure. To achieve this goal, the VG was constructed for two cases: for a representative preprocessed fNIRS time series and for its randomly shuffled version (i.e., the order of appearance of data points in time was randomly shuffled). The PSVG was then estimated from VG for each case. Figure 4(a) shows an example of a time series (a $\Delta [{\mathrm{HbO}}_{2}]$ signal associated with one EC block) and its associated power-law tail of the degree distribution. The shuffled version of this time series and its associated power-law tail of the degree distribution are shown in Fig. 4(b). It can be seen that the degree distribution in the two cases decays at different rates.

The estimated PSVG for the original time series [Fig. 4(a)], ${\mathrm{PSVG}}_{\mathrm{orig}}$, is 3.512. For the shuffled version, random shuffling was performed for 100 rounds, and for each round, the power of scale-freeness, ${\mathrm{PSVG}}_{\mathrm{shuf}}$, was estimated. The averaged ${\mathrm{PSVG}}_{\mathrm{shuf}}$ is obtained as 3.996 [standard deviation is 0.120, range [3.787 to 4.283], refer to Fig. 4(c)]. It is observed that although both the original time series and its randomly shuffled version have the same distribution in terms of amplitude of data points, they have different PSVG values. This result implies that the temporal structure of the time series, rather than the distribution of amplitude of data points, can be characterized from the PSVG values.

**Fig. 5:**
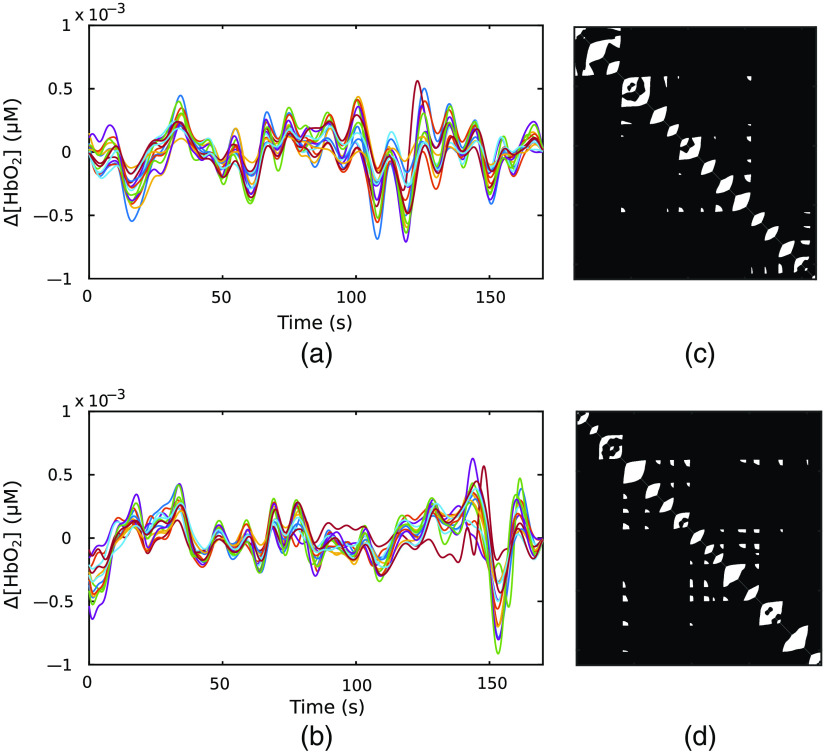
Post-preprocessed $\Delta [{\mathrm{HbO}}_{2}]$ time series recorded from one representative subject under (a) EC condition and (b) GNG condition. The waveforms with different colors represent the time series recorded from different channels. (c) and (d) Adjacent matrices of VGs associated with channel 1 under the two conditions shown in (a) and (b), respectively. The dark color represents that there is no connection (no visibility), and the light color represents the existence of a link (visibility).

Next, the VGs were then constructed for the time series associated with each channel, subject, condition, and block separately. As an example, Figs. 5(a) and 5(b) show two representative $\Delta [{\mathrm{HbO}}_{2}]$ signals recorded from one subject under EC and GNG blocks, respectively. The waveforms with different colors represent the $\Delta [{\mathrm{HbO}}_{2}]$ time series recorded from different channels. Figures 5(c) and 5(d) illustrate two adjacency matrices of VGs associated with EC and GNG from channel 1, respectively. It can be seen that there exist differences in the number of links of the two matrices, indicative of differences in temporal structure of the two time series.

**Fig. 6:**
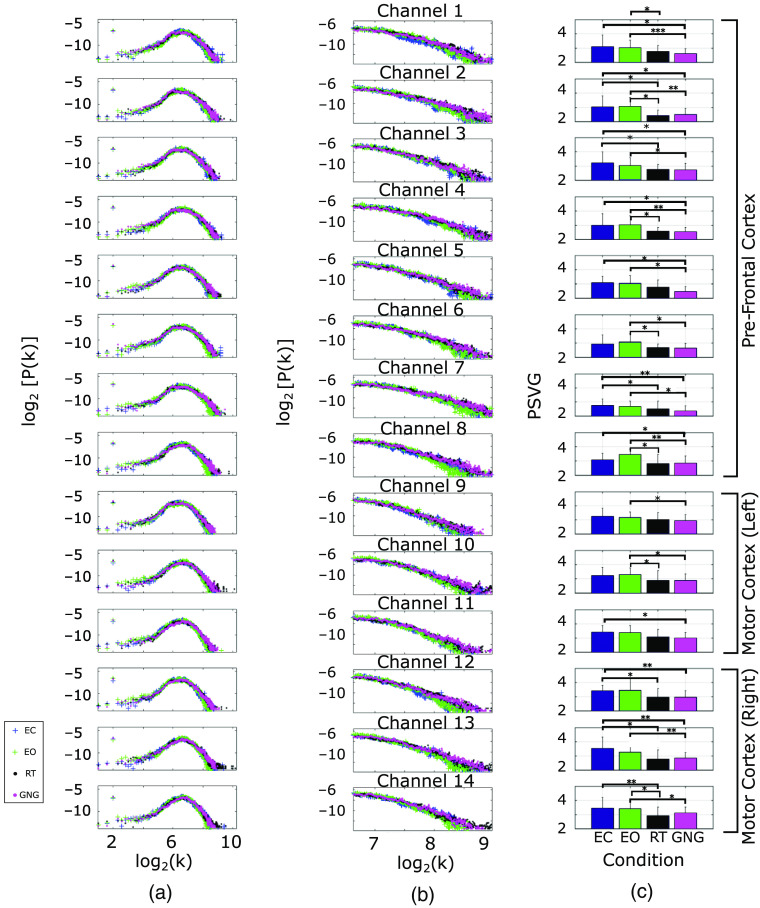
(a) Degree distribution results averaged across subjects for 14 channels. (b) The linear range of the averaged degree distribution for 14 channels. (c) The mean and standard deviation of estimated PSVG for each condition across subjects. The pairs of conditions presenting statistically significant difference are labeled using * notations. *p<0.05, **p<0.01, and ***p<0.001.

To further quantify these differences, we extracted the graph measure of PSVG for each case. Figure 6(a) presents the degree distribution patterns averaged across subjects for each of the 14 channels. The colors correspond to different conditions (EC, EO, RT, and GNG). The zoomed-in power-law tail of the distributions is shown in Fig. 6(b). As can be seen, for majority of channels, the slopes are different for resting-state and task-based time-series.

A least-square regression line was fitted to the power-law tail and from it, the PSVG (equal to the negative of the slope) was calculated ($\gamma =-\frac{\log[P(k)]}{\log(k)}$). The mean and standard deviation of the estimated PSVG values associated with each condition and each channel are presented as bar plots in Fig. 6(c).

To evaluate if the observed differences between PSVG values across conditions are statistically significant, we first performed a one-way repeated analysis of variance (ANOVA). Results are summarized in Table 1. The results from ANOVA with majority of channels showing p<0.05 further motivated us to perform paired-sample student’s t-tests for each channel across each pair of conditions (EO–EC, EO–RT, EO–GNG, EC–RT, EC–GNG, RT–GNG). For each channel, the pairs of conditions that result in significant difference were identified and labeled using “star” notations in Fig. 6(c). The results of this statistical test are also summarized in Table 2. It is shown clearly that for all subjects, the PSVG values associated with resting states (EC and EO) are larger than those associated with task-based (RT and GNG) conditions. Using this result, we hypothesize that the PSVG values of VGs can be used to distinguish brain states. In the following, we perform classification to test this hypothesis.

**Table 1:** Results for one-way repeated ANOVA for each channel.

Channel #	1	2	3	4	5	6	7
F(3,24) #	3.3365	5.9108	2.7622	3.8290	3.5349	2.9006	3.1761
p-Value #	0.0362	0.0036	0.0640	0.0226	0.0299	0.0557	0.0424
Channel #	8	9	10	11	12	13	14
F(3,24) #	4.3358	1.3423	2.7736	1.7232	1.9111	5.2257	3.3787
p-Value #	0.0141	0.2841	0.0633	0.1890	0.1547	0.0064	0.0347

**Table 2:** The p values for the results of the pairwise t-test on PSVG values for various pair conditions, for each channel, and across subjects.

Channel #	1	2	3	4	5	6	7
EC versus EO	0.3681	0.5787	0.2369	0.6743	0.2886	0.7672	0.2388
EC versus RT	0.0713	0.0218*	0.0458*	0.0712	0.0688	0.1134	0.0132*
EC versus GNG	0.0310*	0.0221*	0.0458*	0.0376*	0.0201*	0.1246	0.0081**
EO versus RT	0.0458*	0.0055*	0.0654	0.0317*	0.0696	0.0144*	0.1817
EO versus GNG	0.0002***	0.0019**	0.0345*	0.0068**	0.0323*	0.0121*	0.0298*
RT versus GNG	0.1137	0.6943	0.3728	0.2976	0.1041	0.5075	0.1790
Channel #	8	9	10	11	12	13	14
EC versus EO	0.9388	0.2606	0.7849	0.4129	0.5641	0.1247	0.5024
EC versus RT	0.0957	0.1905	0.0719	0.0549	0.0294*	0.0147*	0.0062**
EC versus GNG	0.0197*	0.0557	0.0671	0.0206*	0.0075**	0.0054**	0.1107
EO versus RT	0.0247*	0.2722	0.0260*	0.1127	0.1309	0.0502	0.0216*
EO versus GNG	0.0085**	0.0232*	0.0166*	0.1055	0.0793	0.0016**	0.0414*
RT versus GNG	0.5892	0.2439	0.5750	0.4066	0.4420	0.6736	0.9127

* p<0.05.

** p<0.01.

*** p<0.001.

### 4.3 Classification Results

Binary classification was performed to evaluate the feasibility of using PSVG in distinguishing brain states. The individual multichannel PSVG values associated with EC and EO conditions were pooled to form the resting-state sample data, and those PSVG values associated with RT and GNG conditions were pooled to form the task-execution sample data. Before pooling, the individual PSVG values were normalized across conditions, so their $\ell_{2}$-norm was equal to 1 for each channel. This procedure left 18 data for each binary condition.

For the classifiers, we chose kNN, linear and nonlinear support vector machine (SVM), linear discriminant analysis (LDA), and quadratic discriminant analysis (QDA). We note that previous studies have used artificial neural networks (ANN) for classification of fNIRS data,^75^ but considering the small sample size in this study, we did not implement ANN.

Given the small sample size here, leave-two-out-cross-validation procedure was used, in which the classification was repeated $(\begin{array}{c} 36\\ 2 \end{array})=630$ times, where, in each time, two samples were assigned to the testing dataset and the remaining samples were assigned to the training dataset. The classification results were evaluated using the measures of accuracy, sensitivity, and specificity. Next, the results were averaged across all repetitions for a given selected number of channels. For kNN, k was selected in the range of 1 to 6. For SVM, different kernels [linear, radial basis function (RBF), quadratic, third and fourth polynomial] were implemented. For discriminant analysis (DA), LDA and QDA were used.

The results for accuracy, specificity, and sensitivity are summarized in Table 3. The best result for kNN is achieved for k=5. The linear SVM performs better than nonlinear SVM. For DA, the performance of LDA is better than QDA. It is observed that the nonlinear classifiers (QDA and nonlinear SVM) did not improve the accuracy of classification due to the relatively small sample size. Overall, it can be seen that kNN, SVM, and LDA have shown significantly better accuracy than that of the chance level (50%). These results reveal that the PSVGs of hemodynamic signals measured from both prefrontal and motor cortices carry discriminatory information for differentiating resting-state and task-execution conditions.

**Table 3:** Classification performance results obtained for different classifiers (kNN, SVM, and DA when considering all channels. For each classifier, the bold values signify the best obtained accuracy.

Classifier	kNN						SVM					DA	
k, Kernel	1	2	3	4	5	6	Linear	RBF	Quadratic	Third polynomial	Fourth polynomial	LDA	QDA
Accuracy (%)	61	64	80	78	**81**	80	**80**	77	71	70	54	**73**	23
Sensitivity (%)	72	50	89	83	94	88	88	84	77	72	55	79	18
Specificity (%)	51	78	72	72	67	73	72	69	64	69	53	66	27

## 5 Discussions and Conclusions

The human brain is a highly complex system with nonlinear dynamical behavior.^76^ As such, methods such as fractal analysis, which can reveal nonlinearities in the recordings associated with brain activities, can serve as an important complementary approach to the commonly used methods (e.g., used for functional localization or functional connectivity) in order to gain a more comprehensive knowledge about how the brain functions.

In this study, we investigated the fractality of fNIRS-recorded time series obtained during task execution or when the brain is at rest using VG. Fractal dimension for all conditions and recordings was first estimated through SWV analysis. It was shown that fNIRS recordings, regardless of the experimental conditions, exhibit high fractality. This result is in line with previous studies that used EEG or fMRI to monitor brain function.^42^^,^^77^^,^^78^

Next, VGs were constructed for all fNIRS time series recordings and their corresponding PSVG values were calculated. Results showed that for most channels, the difference in PSVG values for cases when the brain is at rest and when the brain is engaged in executing tasks is statistically significant. To the best of our knowledge, this is the first study that explores the possibility of employing VG in differentiating rest and task-execution brain states.

The capability of VG-based metrics in differentiating brain states was further examined by performing classification using the PSVG values estimated during rest or task execution as features to the classifier. The feature dimension was equal to the number of channels. A wide range of classifiers was used and although the number of training and testing samples in this study was small, a reasonably good accuracy was obtained.

One application of VG-based metrics being used as features would be to use them as biomarkers for diagnosing brain-related disorders.^38^^–^^40^ Due to the instrumental advantages of fNIRS technology (e.g., portability and low cost), fNIRS has great potential for clinical settings. As such, finding features in fNIRS recordings that can serve as biomarkers for diagnosis are of great interest to clinicians. The results of this work show the capability of VG-based metrics in finding features that are based on nonlinear properties of fNIRS recordings, which could have applications in clinical settings, for example, in cases, where disease-specific information exist in the nonlinear temporal characteristics of the recordings.

In future studies, we plan to investigate how other metrics of VG, such as closeness and betweenness centrality, clustering coefficient, and transitivity,^49^^,^^63^ can characterize the temporal structures of fNIRS recordings. Additionally, we plan to extend VG to multilayer VG, where relations among time series of different channels can also be quantitatively characterized,^79^ providing more interesting information about how the brain functions.
